# 超高效液相色谱-串联质谱法同时测定禽蛋中31种产蛋期禁用兽药

**DOI:** 10.3724/SP.J.1123.2023.11015

**Published:** 2024-05-08

**Authors:** Wanyan ZHU, Hongwei ZHANG, Lizhi CHE, Wenyuan XU, Caizhi LUN, Jiufei XU, Hao XU, Wei CHEN

**Affiliations:** 1.临沂海关综合技术服务中心, 山东 临沂 276034; 1. Comprehensive Technology Center of Linyi Customs, Linyi 276034, China; 2.青岛海关技术中心, 山东 青岛 266109; 2. Technology Center of Qingdao Customs, Qingdao 266109, China

**Keywords:** 超高效液相色谱-串联质谱, QuEChERS, 兽药, 禽蛋, ultra performance liquid chromatography-tandem mass spectrometry (UPLC-MS/MS), QuEChERS, veterinary drugs, poultry egg

## Abstract

采用改良的QuEChERS法进行前处理,建立了超高效液相色谱-串联质谱同时测定禽蛋(鸡蛋、鹅蛋、鸭蛋)中9类31种产蛋期禁用兽药(大环内酯类、解热镇痛类、磺胺类、抗菌增效类、抗球虫类、抗线虫类、喹诺酮类、四环素类、酰胺醇类)的检测方法,并对样品前处理和色谱条件进行了优化。称取2.00 g样品,先加入2 mL 0.1 mol/L 乙二胺四乙酸二钠溶液,涡旋1 min,将样品充分分散,再用8 mL 3%乙酸乙腈提取,氯化钠(2 g)盐析,离心后,上清液采用十八烷基硅烷键合硅胶(C_18_, 100 mg)、*N*-丙基乙二胺(PSA, 50 mg)和氨基(NH_2_, 50 mg)吸附剂联合净化,待测分析物经Waters CORTECS UPLC C_18_色谱柱 (150 mm×2.1 mm,1.8 μm)分离,分别在正、负离子模式下采集,空白基质匹配标准曲线外标法定量。结果表明,31种产蛋期禁用兽药在相应浓度范围内具有良好的线性关系,相关系数>0.99,检出限为0.3~3.0 μg/kg,定量限为1.0~10.0 μg/kg。在定量限、最大残留限量、2倍最大残留限量3个添加水平下,3种禽蛋基质中31种产蛋期禁用兽药的平均加标回收率为61.2%~105.7%,相对标准偏差为1.8%~17.6%(*n*=6)。利用所建立的方法对30份禽蛋样品(20份鸡蛋、5份鹅蛋和5份鸭蛋)进行检测,共有1份样品检出恩诺沙星。本方法简单、经济、实用,适用于禽蛋中多类产蛋期禁用兽药的同时测定。

## Abstract (en)

The consumption of poultry eggs has increased in recent years owing to the abundance of production and improvements in living standards. Thus, the safety requirements of poultry eggs have gradually increased. At present, few reports on analytical methods to determine banned veterinary drugs during egg-laying period in poultry eggs have been published. Therefore, establishing high-throughput and efficient screening methods to monitor banned veterinary drugs during egg-laying period is imperative. In this study, an analytical method based on ultra performance liquid chromatography-tandem mass spectrometry (UPLC-MS/MS) combined with QuEChERS-based techniques was developed for the simultaneous determination of 31 banned veterinary drugs encompassing nine drug classes (macrolides, antipyretic and analgesic drugs, sulfonamides, antibacterial synergists, anticoccidials, antinematodes, quinolones, tetracyclines, amphenicols) in different types of poultry eggs. The main factors affecting the response, recovery, and sensitivity of the method, such as the extraction solvent, purification adsorbent, LC separation conditions, and MS/MS parameters, were optimized during sample pretreatment and instrumental analysis. The 31 veterinary drug residues in 2.00 g eggs were extracted with 2 mL of 0.1 mol/L ethylene diamine tetraacetic acid disodium solution and 8 mL 3% acetic acid acetonitrile solution, and salted out with 2 g of sodium chloride. After centrifugation, 5 mL of the supernatant was cleaned-up using the QuEChERS method with 100 mg of octadecylsilane-bonded silica gel (C_18_), 50 mg of *N*-propylethylenediamine (PSA), and 50 mg of NH_2_-based sorbents. After nitrogen blowing and redissolution, the 31 target analytes were separated on a Waters CORTECS UPLC C_18_ analytical chromatographic column (150 mm×2.1 mm, 1.8 μm) at a flow rate, column temperature, and injection volume of 0.4 mL/min, 30 ℃, and 5 μL, respectively. Among these analytes, 26 analytes were acquired in dynamic multiple reaction monitoring (MRM) mode under positive electrospray ionization (ESI^+^) conditions using (A) 5 mmol/L ammonium acetate (pH 4.5) and (B) acetonitrile as mobile phases. The gradient elution program was as follows: 0-2.0 min, 12%B-30%B; 2.0-7.5 min, 30%B-50%B; 7.5-10.0 min, 50%B; 10.0-10.1 min, 50%B-100%B; 10.1-12.0 min, 100%B; 12.0-12.1 min, 100%B-12%B; The five other target analytes were acquired in MRM mode under negative electrospray ionization (ESI^-^) conditions using (A) H_2_O and (B) acetonitrile as mobile phases. The gradient elution program was as follows: 0-2.0 min, 12%B-40%B; 2.0-6.0 min, 40%B-80%B; 6.0-6.1 min, 80%B-100%B; 6.1-8.0 min, 100%B; 8.0-8.1 min, 100%B-12%B. Matrix-matched external standard calibration was used for quantification. The results showed that all the compounds had good linear relationships within their respective ranges, with correlation coefficients of >0.99. The limits of detection (LODs) and quantitation (LOQs) were 0.3-3.0 μg/kg and 1.0-10.0 μg/kg, respectively. The average recoveries of the 31 banned veterinary drugs spiked at three levels (LOQ, maximum residue limit (MRL), and 2MRL) in poultry eggs ranged from 61.2% to 105.7%, and the relative standard deviations (RSDs) ranged from 1.8% to 17.6%. The developed method was used to detect and analyze banned veterinary drugs in 30 commercial poultry egg samples, including 20 eggs, 5 duck eggs, and 5 goose eggs. Enrofloxacin was detected in one egg with a content of 12.3 μg/kg. The proposed method is simple, economical, practical, and capable of the simultaneous determination of multiple classes of banned veterinary drugs in poultry eggs.

禽蛋因营养价值高,含有人体需要的优质蛋白和其他丰富的营养元素,在很早之前就被人们作为一种营养品食用。近年来,随着物质的丰富、人们生活水平的提高,禽蛋的食用量日益增加,国人对禽蛋的安全要求也逐步提升。为提高禽蛋产量,个别禽类饲养者不遵守产蛋期的喂养规定,私自添加使用产蛋期禁用兽药,造成禽蛋中的兽药残留超标,损害人体健康^[1,2]^。GB 31650-2019 《食品安全国家标准 食品中兽药最大残留限量》^[3]^明确规定一些兽药在家禽产蛋期禁用,并且在GB 31650.1-2022《食品安全国家标准 食品中41种兽药最大残留限量》^[4]^中进一步规定了产蛋期禁用兽药在禽蛋中的最大残留限量(MRL),表1列出了31种产蛋期禁用兽药的药物名称、药物种类及最大残留限量。这31种兽药或为已批准动物源性食品中最大残留限量规定的兽药,或为允许用于食品动物且不需要制定残留限量的兽药,但均为家禽产蛋期禁用兽药,这说明国家对家禽产蛋期的用药控制更为严格,对禽蛋中兽药残留检测方法的性能指标要求更高。目前,专门检测家禽产蛋期禁用兽药残留的方法报道还较少,因此建立禽类产蛋期禁用兽药残留的检测方法十分必要。

**表1:** 31种兽药的类别、名称及最大残留限量

Class	Name	MRL^*^/ (μg/kg)
Antinematodes	levamisole (左旋咪唑)	5
Antibacterial	trimethoprim (甲氧苄啶)	10
synergists		
Macrolides	tilmicosin (替米考星)	10
Quinolones	danofloxacin (达氟沙星)	10
	enrofloxacin (恩诺沙星)	10^1)^
	ciprofloxacin (环丙沙星)	
	flumequine (氟甲喹)	10
	oxolinic acid (恶喹酸)	10
	sarafloxacin (沙拉沙星)	5
	lomefloxacin (洛美沙星)	2
	norfloxacin (诺氟沙星)	2
	ofloxacin (氧氟沙星)	2
	pefloxacin (培氟沙星)	2
Tetracyclines	doxycycline (多西环素)	10
Sulfonamides	sulfadimidine (磺胺二甲嘧啶)	10
	sulfadiazine (磺胺嘧啶)	10^2)^
	sulfathiazole (磺胺噻唑)	
	sulfamerazine (磺胺甲基嘧啶)	
	sulfameter (磺胺甲氧嘧啶)	
	sulfamonomethoxine	
	(磺胺间甲氧嘧啶)	
	sulfachloropyridazine (磺胺氯哒嗪)	
	sulfadoxine (磺胺邻二甲氧嘧啶)	
	sulfadimethoxine (磺胺二甲氧嘧啶)	
	sulfamethoxazole (磺胺甲基异噁唑)	
	sulfaquinoxaline (磺胺喹噁啉)	
Amphenicols	thiamphenicol (甲砜霉素)	10
	florfenicol-amine (氟苯尼考胺)	10^3)^
	florfenicol (氟苯尼考)	
Anticoccidials	toltrazuril sulfone (托曲珠利砜)	10
	diclazuril (地克珠利)	10
Antipyretic and	aspirin (阿司匹林)	10
analgesic drugs		

* From the limit regulations in GB 31650.1-2022. 1) total of enrofloxacin and ciprofloxacin; 2) total of other sulfonamides; 3) total of florfenicol-amine and florfenicol.

随着超高效液相色谱-串联质谱仪(UPLC-MS/MS)的普及,加之该设备具有灵敏度高、定性定量准确的特点,UPLC-MS/MS已成为兽药残留测定的首选技术方法^[5⇓⇓⇓⇓-10]^。QuEChERS前处理方法具备简单、快速、成本低的特点,日益受到兽药残留检测人员的青睐^[11⇓⇓⇓⇓-16]^。利用超高效液相色谱-串联质谱法进行鸡蛋中多药物残留分析虽有报道,但存在分析药物种类不全、定量限高、基质单一等问题。例如,方从容等^[12]^报道的鸡蛋中多兽药残留检测方法不包含解热镇痛类、抗球虫类等产蛋期禁用兽药,且强力霉素、沙拉沙星、诺氟沙星、培氟沙星、洛美沙星、恩诺沙星及氧氟沙星的检出限均高于GB 31650.1-2022中的最大残留限量要求。陈兴连等^[17]^报道的鸡蛋中多兽药残留检测方法不包含大环内酯类、解热镇痛类、抗菌增效类、抗球虫类、抗线虫类、四环素类产蛋期禁用兽药,且培氟沙星、洛美沙星的定量限高于GB 31650.1-2022中的最大残留限量要求。Wang等^[18]^报道的鸡蛋中多残留检测方法不包含大环内酯类、解热镇痛类、抗球虫类、抗线虫类、四环素类等产蛋期禁用兽药。此外,现有检测方法检测样品多为鸡蛋,检测基质比较单一。本工作采用QuEChERS前处理方法,建立了禽蛋中31种共9大类产蛋期禁用兽药的UPLC-MS/MS检测方法(其中,因阿司匹林的活性成分是乙酰水杨酸,参照标准SN/T 1922-2007^[19]^,本工作实际检测的是阿司匹林的代谢产物水杨酸),以期为禽蛋中产蛋期禁用兽药的监督提供技术保障。

## 1 实验部分

### 1.1 仪器、材料与试剂

Agilent 1290-6470型超高效液相色谱-串联质谱仪(美国Agilent公司),配有电喷雾离子源(ESI); CR22GⅢ型高速冷冻离心机(日本HITACHI公司); ULTRA-TURRAX T25型均质器、GENIUS 3涡旋混匀器和HS260型多用调速振荡器(德国IKA公司); Caliper TurboVap LV型氮吹仪(美国Caliper公司)。

中性氧化铝、*N*-丙基乙二胺(PSA)、十八烷基硅烷键合硅胶(C_18_)、石墨化炭黑(GCB)、氨基(NH_2_)吸附剂均购于天津Bonna-Agela公司。

乙腈(色谱纯,美国MREDA公司);甲醇(色谱纯,美国LabServ公司);甲酸(色谱纯,天津市光复精细化工研究所);氯化钠(分析纯,天津博迪化工股份有限公司);乙二胺四乙酸二钠(EDTA)(分析纯,国药集团化学试剂有限公司);实验用水为由Milli-Q IQ7000纯水机(德国Merck Millipore公司)制得的超纯水。

地克珠利、托曲珠利砜、水杨酸标准物质购自北京曼哈格生物科技有限公司,纯度均大于99.4%;其他28种兽药标准储备液均购自农业部环境保护科研监测所,质量浓度均为100 mg/L。

### 1.2 标准溶液的配制

分别准确称取适量地克珠利、托曲珠利砜、水杨酸标准物质,用甲醇溶解定容,配制成质量浓度为100 mg/L的标准储备液,于-18 ℃下避光保存。其中,地克珠利和托曲珠利砜标准储备液有效期12个月,水杨酸标准储备液有效期3个月。

分别吸取0.25 mL左旋咪唑、沙拉沙星标准储备液, 0.1 mL洛美沙星、诺氟沙星、氧氟沙星、培氟沙星标准储备液, 0.5 mL其余兽药的标准储备液于50 mL容量瓶中,用甲醇定容至刻度,即得对应质量浓度分别为0.5、0.2、1.0 mg/L的31种兽药混合标准中间溶液。

### 1.3 样品前处理

称取2.00 g样品,置于50 mL离心管中,加入2 mL 0.1 mol/L EDTA溶液,涡旋1 min,将样品充分分散,再加入2 g氯化钠和8 mL 3%乙酸乙腈,振荡提取30 min, 10000 r/min离心5 min,取5 mL上清液待净化。

将5 mL上清液加入含有100 mg C_18_、50 mg PSA和50 mg NH_2_吸附剂的15 mL离心管中,涡旋1 min, 10000 r/min离心5 min,再取4 mL上清液于另一离心管中,40 ℃氮吹至干,用1 mL乙腈-0.1%甲酸水溶液(1∶9, v/v)复溶,过0.22 μm滤膜,进行UPLC-MS/MS测定。

### 1.4 仪器工作条件

色谱条件 Waters CORTECS UPLC C_18_色谱柱(150 mm×2.1 mm, 1.8 μm);柱温:30 ℃;流速:0.4 mL/min;进样量:5 μL;正离子模式流动相:A为5 mmol/L乙酸铵(pH 4.5), B为乙腈;梯度洗脱程序:0~2.0 min, 12%B~30%B; 2.0~7.5 min, 30%B~50%B; 7.5~10.0 min, 50%B; 10.0~10.1 min, 50%B~100%B; 10.1~12.0 min, 100%B; 12.0~12.1 min, 100%B~12%B。负离子模式流动相:A为水,B为乙腈;梯度洗脱程序:0~2.0 min, 12%B~40%B; 2.0~6.0 min, 40%B~80%B; 6.0~6.1 min, 80%B~100%B; 6.1~8.0 min, 100%B; 8.0~8.1 min, 100%B~12%B。

质谱条件 电喷雾离子源;动态多反应离子监测模式(dynamic MRM);干燥气温度:300 ℃;干燥气流速:12 L/min;雾化气压力:241 kPa;鞘气温度:300 ℃;鞘气流速:12 L/min;正离子模式下毛细管电压:3500 V,喷嘴电压:0 V;负离子模式下毛细管电压:-3000 V,喷嘴电压:-500 V; 31种目标化合物的保留时间、母离子、子离子、毛细管出口电压、碰撞能等参数见表2。

**表2:** 31种兽药的参考保留时间、质谱参数、检出限和定量限

Drug	t_R_/min	ESI	Parent ion (m/z)	Product ions (m/z)	Fragmentor/V	CEs/eV	LOD/(μg/kg)	LOQ/(μg/kg)
Levamisole	2.28	+	205.3	178.1^*^, 91.2	100	20, 45	0.6	2.0
Trimethoprim	2.51	+	291.4	230.2^*^, 123.2	120	25, 30	0.9	3.0
Tilmicosin	4.01	+	870.0	174.0^*^, 132.1	200	50, 50	0.9	3.0
Danofloxacin	2.79	+	358.3	340.3^*^, 82.0	100	25, 42	0.9	3.0
Enrofloxacin	2.99	+	360.0	245.0^*^, 316.0	120	27, 27	0.9	3.0
Ciprofloxacin	2.62	+	332.0	314.0^*^, 231.0	120	25, 25	0.9	3.0
Flumequine	5.92	+	262.3	244.2^*^, 202.1	110	20, 40	0.9	3.0
Oxolinic acid	4.27	+	262.3	244.1^*^, 216.0	100	20, 35	0.9	3.0
Sarafloxacin	3.22	+	385.9	299.0^*^, 342.1	100	15, 25	0.9	3.0
Lomefloxacin	2.77	+	352.0	265.0^*^, 308.3	120	10, 20	0.6	2.0
Norfloxacin	2.58	+	320.0	302.1^*^, 233.0	120	24, 24	0.3	1.0
Ofloxacin	2.62	+	362.0	261.0^*^, 318.0	120	26, 26	0.3	1.0
Pefloxacin	2.74	+	334.0	233.0^*^, 290.1	100	10, 20	0.6	2.0
Doxycycline	3.41	+	445.0	428.2^*^, 154.0	120	27, 40	3.0	10.0
Sulfadimidine	3.10	+	279.0	186.0^*^, 156.3	120	22, 20	0.9	3.0
Sulfadiazine	2.32	+	251.0	156.0^*^, 108.3	80	16, 22	0.9	3.0
Sulfathiazole	2.46	+	256.0	156.0^*^, 108.1	80	15, 25	0.9	3.0
Sulfamerazine	2.76	+	265.0	156.0^*^, 172.1	120	18, 20	0.9	3.0
Sulfameter	3.14	+	281.0	156.0^*^, 215.1	100	18, 18	0.9	3.0
Sulfamonomethoxine	3.44	+	281.0	156.0^*^, 126.1	120	20, 25	0.9	3.0
Sulfachloropyridazine	3.60	+	285.7	156.0^*^, 108.1	80	12, 22	0.9	3.0
Sulfadoxine	3.80	+	311.0	156.0^*^, 108.2	140	25, 30	0.9	3.0
Sulfadimethoxine	4.74	+	311.0	156.0^*^, 108.0	140	25, 30	0.9	3.0
Sulfamethoxazole	3.91	+	254.0	156.0^*^, 108.1	100	15, 25	0.9	3.0
Sulfaquinoxaline	4.70	+	301.0	156.0^*^, 108.1	80	15, 27	0.9	3.0
Florfenicol-amine	1.32	+	247.9	230.1^*^, 130.0	110	10, 15	0.9	3.0
Thiamphenicol	2.65	-	354.0	290.0^*^, 185.2	110	10, 25	0.3	1.0
Florfenicol	3.38	-	356.0	336.0^*^, 185.1	100	10, 15	0.3	1.0
Toltrazuril sulfone	5.72	-	456.5^* #^		120	0	1.5	5.0
Diclazuril	6.05	-	407.0	336.0	120	20	1.5	5.0
			405.0	334.0^*^	120	20		
Salicylic acid	2.17	-	137.0	93.0^*^, 65.3	80	15, 35	0.9	3.0

* Quantitative ion. Salicylic acid: metabolites of aspirin. # It is difficult to obtain suitable product ions of toltrazuril sulfone, so the parent ion was used for quantification.

### 1.5 基质效应(ME)的计算

基质是指样品提取液中除待测分析物以外的物质。基质共提物与目标分析物在ESI源中会发生竞争电离,从而出现干扰目标化合物的质谱信号,影响目标化合物响应强度的现象,即为基质效应。基质效应影响定量分析结果的准确性和可靠性,是方法建立过程中需要考察的一个重要参数。本工作以ME=(基质标准曲线的斜率/溶剂标准曲线的斜率-1)×100%的值来表示基质效应的大小,负值表示存在抑制效应,正值表示存在增强效应,当比值在-20%~20%时,表示基质效应不明显^[17,20]^。

## 2 结果与讨论

### 2.1 前处理条件的优化

#### 2.1.1 提取溶液的选择

兽药残留常用的提取溶剂有甲醇、乙腈、乙酸乙酯,相比之下,3种溶剂中甲醇的极性最强,渗透组织的能力也更好,但提取的极性杂质也更多,此外甲醇去除蛋白质时形成的是絮状沉淀,悬浮于提取溶液中,不利于后续的净化;乙酸乙酯的极性低,渗透组织的能力差,对多类药物同时提取的提取效率不高;乙腈去除蛋白质、脂肪等的沉淀效果好,对多数目标物的提取效率高。综合3种溶剂的提取特点,结合目标基质,本工作选择乙腈作为提取溶剂。

本工作选取3种样品中基质最为复杂、预实验中基质效应最强的鸭蛋作为前处理优化的样品基质,以ME和提取回收率作为判定依据,考察了乙腈、水-乙腈、0.1 mol/L EDTA溶液-乙腈、0.1 mol/L EDTA溶液-3%乙酸乙腈4种提取溶液的提取效果(加入提取溶液时,先加水相,涡旋1 min后,再加有机相)。具体方法:分别用4种提取溶剂按照1.3节步骤提取、净化不含有待测兽药的鸭蛋样品,获得空白基质溶液,配制系列基质标准溶液,同时配制相同浓度的系列溶剂标准溶液,进样测定,计算其各自的ME,结果见表3;在不含有待测兽药的鸭蛋样品中添加标准溶液,获得目标分析物浓度相同的添加样品,用4种提取溶剂按相同的步骤提取、净化,进样测定,再用各自的基质标准曲线校正,计算每类药物的平均回收率,结果见图1。

**表3:** 不同提取溶液对31种兽药基质效应的影响

Drug	Acetonitrile	H_2_O- acetonitrile	0.1 mol/L EDTA- acetonitrile	0.1 mol/L EDTA-acetonitrile (containing 3% acetic acid)
Levamisole	-73.4	-46.8	-42.4	-44.7
Trimethoprim	-63.7	-53.6	-51.4	-50.2
Tilmicosin	56.3	68.9	76.2	73.5
Danofloxacin	-45.5	-30.5	-34.1	-35.9
Enrofloxacin	-48.7	-21.3	-13.5	-15.0
Ciprofloxacin	-62.3	-52.2	-49.5	-47.4
Flumequine	-37.8	-17.4	-14.6	-12.4
Oxolinic acid	30.3	18.9	25.7	26.5
Sarafloxacin	-42.0	-50.0	-48.7	-54.3
Lomefloxacin	-14.3	-17.1	-16.2	-18.7
Norfloxacin	-53.3	-41.4	-37.8	-39.8
Ofloxacin	-19.2	-13.5	-16.7	-17.3
Pefloxacin	-42.7	-32.5	-28.8	-27.1
Doxycycline	35.5	38.7	30.6	26.6
Sulfadimidine	-30.8	-27.6	-32.4	-35.0
Sulfadiazine	12.4	8.9	4.6	0
Sulfathiazole	-33.4	-28.7	-33.9	-37.6
Sulfamerazine	-40.5	-36.4	-42.0	-44.0
Sulfameter	-23.2	-13.6	-16.7	-18.8
Sulfamonomethoxine	-20.9	-16.2	-15.6	-14.6
Sulfachloropyridazine	-15.1	-8.4	-10.4	-9.1
Sulfadoxine	-13.8	-15.3	-14.1	-13.0
Sulfadimethoxine	16.7	13.8	16.4	17.6
Sulfamethoxazole	-15.4	-12.3	-14.5	-10.9
Sulfaquinoxaline	23.6	-8.7	-6.7	-3.9
Florfenicol-amine	-60.6	-57.0	-64.0	-72.0
Thiamphenicol	-15.2	-14.7	-12.6	-16.3
Florfenicol	-18.9	-12.6	-11.9	-8.9
Toltrazuril sulfone	-14.4	-16.5	-18.3	-17.8
Diclazuril	-17.6	-15.3	-12.6	-11.0
Salicylic acid	-11.7	-11.7	-13.8	-15.1

**图1:**
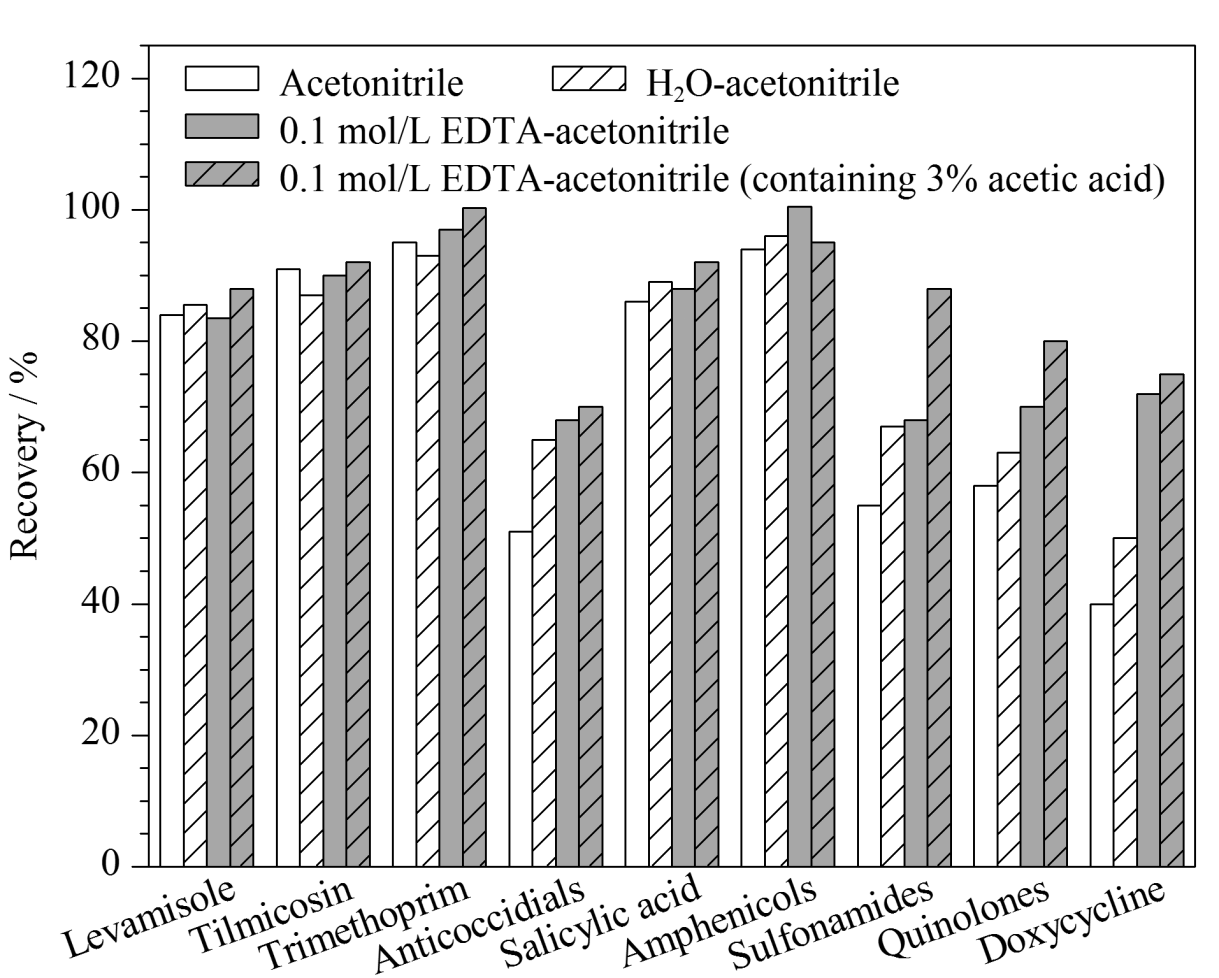
不同提取溶液对每类药物平均回收率的影响

从基质效应看,单独用乙腈提取时基质效应最大,只有12种兽药基质效应不明显,15种兽药存在明显的基质抑制效应,4种兽药存在明显的基质增强效应。在水-乙腈、0.1 mol/L EDTA溶液-乙腈、0.1 mol/L EDTA溶液-3%乙酸乙腈3种提取溶液中,基质效应不明显的兽药数量均为17,表明在这3种提取溶液中,兽药的基质效应相差不大,且明显好于纯乙腈提取溶剂。

从提取回收率看,左旋咪唑、替米考星、甲氧苄啶、水杨酸、酰胺醇类药物在4种提取溶液中回收率相差不大,而其他4类兽药在纯乙腈中的提取回收率普遍低于其他3种提取体系(低10%~15%),这可能是因为在提取前,加入水相,使基质充分分散更有利于待测兽药的提取,另外,盐析分层时水相可以带出部分水溶性杂质,降低基质干扰,各待测兽药在乙腈提取溶剂中的基质效应高于其他3种体系的基质效应也说明了这一点。水相中不加EDTA时,强力霉素的回收率不高于40%,加入2 mL 0.1 mol/L EDTA溶液后,强力霉素的回收率达到65%以上,喹诺酮类药物的回收率也有所增加,这是由于强力霉素和喹诺酮类药物易与金属离子络合,加入EDTA溶液后,金属离子优先与EDTA发生络合反应,从而将强力霉素和喹诺酮类药物释放出来。酸化乙腈有利于磺胺、喹诺酮类化合物分配在乙腈中,提取回收率都有所提高,磺胺类药物的回收率提高20%左右。综合考虑所有待测兽药,本工作选用0.1 mol/L EDTA溶液-3%乙酸乙腈(2∶8, v/v)作为提取溶液。

#### 2.1.2 净化剂的选择

本工作选取QuEChERS方法中常用的净化剂C_18_、PSA、NH_2_、中性氧化铝、GCB进行实验。将质量浓度均为1.0 mg/L的31种兽药混合标准中间溶液(见1.2节)稀释为质量浓度均为20 μg/L的31种兽药混合标准溶液,分别加入不同量的C_18_、PSA、NH_2_、中性氧化铝、GCB净化剂,涡旋、离心、过滤后进样测定,通过比较加入净化剂前后待测兽药响应的变化,来判断净化剂对待测兽药的吸附性。结果表明,C_18_对待测兽药基本没有吸附;NH_2_的用量为15 g/L时对待测兽药的吸附可以忽略不计,但随着用量的增加,对磺胺类化合物及地克珠利和托曲珠利砜产生不同程度的吸附,且对喹诺酮类化合物的峰形有干扰;PSA的用量超过30 g/L时,对氟甲喹、恶喹酸和部分磺胺产生不同程度的吸附;中性氧化铝对喹诺酮类化合物和水杨酸吸附严重;GCB对大部分待测兽药吸附严重。

C_18_主要吸附脂肪等非极性干扰物,PSA和NH_2_作用相似,都能够去除极性干扰物质,但随着PSA、NH_2_用量的增加,会对待测兽药产生不同程度的吸附。本研究采用PSA和NH_2_组合使用的方式,这样每种净化剂的用量不大,不会对待测兽药产生吸附,而又尽可能地去除极性干扰物。实验进一步比较了净化剂的用量对净化效果的影响,使用不含待测兽药的空白样品按照所述前处理步骤提取,移取4 mL空白提取液,分别加入不同量的C_18_、PSA、NH_2_进行净化、浓缩、复溶,制备基质标准曲线,计算各自的基质效应。结果表明,C_18_的用量为100 mg和200 mg时,基质效应相差不大,均有13种兽药基质效应不明显(-20%≤ME≤20%);当加入50 mg PSA和50 mg NH_2_后,17种兽药的基质效应不明显,此外氟甲喹的基质效应由95%降到45%,且回收率也能满足分析要求。因此最终选择100 mg C_18_+50 mg PSA+50 mg NH_2_作为净化剂。

### 2.2 色谱条件的选择

本工作比较了不同粒径、不同长度的4种反相色谱柱:Eclipse Plus C_18_ (150 mm×2.1 mm, 3.5 μm)、 Waters CORTECS UPLC C_18_ (150 mm×2.1 mm, 1.8 μm)、 XBridge peptide BEH C_18_ (100 mm×2.1 mm, 3.5 μm)和Eclipse Plus C_18_ (50 mm×2.1 mm, 1.8 μm),同时考察了正离子模式下,乙腈-0.1%甲酸溶液、乙腈-0.1%甲酸溶液(含5 mmol/L乙酸铵)、乙腈-5 mmol/L乙酸铵溶液(pH 4.5)、甲醇-5 mmol/L乙酸铵溶液(pH 4.5)4种流动相体系对31种兽药出峰情况的影响。结果表明,31种待测兽药在Waters CORTECS UPLC C_18_ (150 mm×2.1 mm, 1.8 μm)这款实心核颗粒色谱柱上的峰形好,色谱峰更为尖锐,色谱分离效果更好;当有机相为甲醇时,容易出现前伸峰或拖尾峰,峰形展宽;当有机相为乙腈、水相为5 mmol/L乙酸铵溶液(用乙酸调pH为4.5)时,待测分析物的峰形要好于另外两种有机相为乙腈的流动相体系。此外,负离子模式下,当有机相为乙腈、水相为纯水时,待测分析物的响应均能满足检测需求。因此,最终选定Waters CORTECS UPLC C_18_ (150 mm×2.1 mm, 1.8 μm)为分析柱,乙腈-5 mmol/L乙酸铵(pH 4.5)为正离子模式流动相,乙腈-水为负离子模式流动相。

采用优化的色谱柱和流动相,31种兽药均能在7 min内出峰,所有待测兽药的峰形、响应良好。此外,母离子、子离子均相同的磺胺邻二甲氧嘧啶和磺胺二甲氧嘧啶两种同分异构体也能实现完全分离。空白基质加标样品中31种兽药的MRM色谱图见图2。从图2可以看出,样品基质不干扰待测兽药的测定。

**图2:**
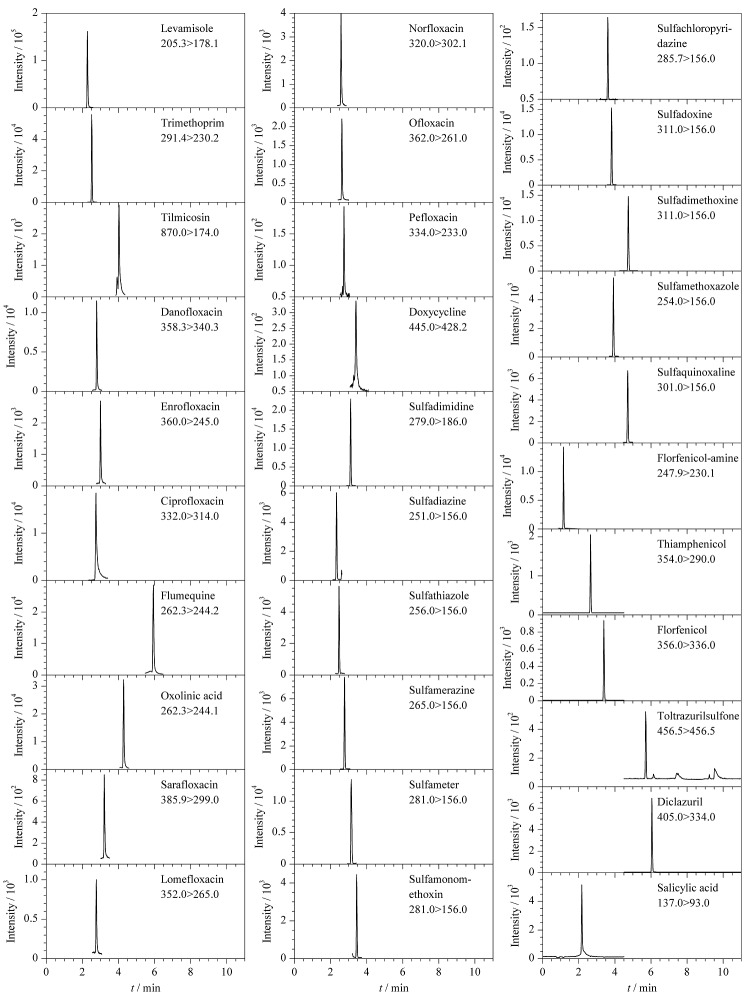
空白基质加标样品中31种兽药的MRM色谱图

### 2.3 基质效应考察

不同样品产生的基质效应可能会不同,本工作考察了鸡蛋、鹅蛋和鸭蛋3种样品的基质效应。选取不含有待测兽药的空白样品,按1.3节方法处理,获得基质空白提取液,配制成相同浓度的系列基质匹配标准溶液,进样测定,分别获得鸡蛋、鹅蛋、鸭蛋的基质匹配标准曲线和溶剂标准曲线,采用1.5节公式计算,31种兽药的基质效应见图3。从图3可看到,31种兽药在不同样品中其基质效应差异较大,鸭蛋的基质效应最大,鸡蛋的基质效应最小。此外,在鸡蛋、鹅蛋、鸭蛋3种样品中基质效应不明显的兽药种类数量分别为22、19、17,其余兽药均存在明显的基质抑制或基质增强效应。为保证实验结果的准确性,本研究选用基质匹配标准曲线外标法定量,最终实验结果表明,此定量方法可以有效消除基质效应的影响。

**图3:**
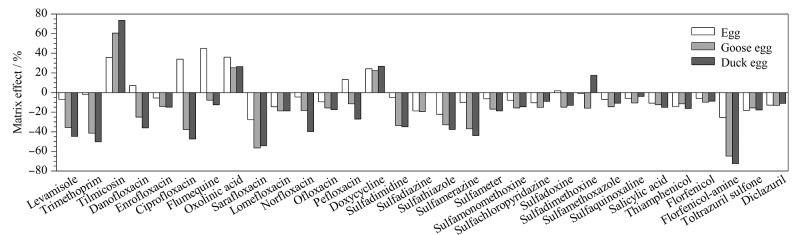
31种兽药的基质效应

### 2.4 方法学考察

#### 2.4.1 线性关系、检出限和定量限

用空白基质提取液配制系列浓度的标准溶液,以待测兽药定量离子的峰面积(*Y*)对质量浓度(*X*, μg/L)绘制标准曲线,得到线性方程及相关系数,31种兽药在各自的线性范围内具有良好的线性关系,相关系数均大于0.99。

因不同样品的基质效应不同,本工作选用基质效应最大的鸭蛋样品作为基质,来确定方法的检出限(LOD)和定量限(LOQ)。在空白鸭蛋样品中添加低浓度待测兽药,进行样品处理后进样测定,取定量离子的信噪比(*S/N*)≥3时的含量作为检出限,取定量离子的*S/N*≥10时的含量作为定量限, 31种兽药的检出限和定量限见表2。从表2可以看到,31种兽药的检出限为0.3~3.0 μg/kg,定量限为1.0~10.0 μg/kg,均小于或等于GB 31650.1-2022中各兽药的最大残留限量。

#### 2.4.2 回收率和精密度

在不含有待测兽药的鸡蛋、鸭蛋、鹅蛋样品中做3个水平(LOQ、MRL、2MRL)的加标回收率试验,每个水平平行做6次,以对应的基质标准曲线定量,计算各待测兽药的平均回收率和相对标准偏差(RSD),结果见表4。31种兽药在3种禽蛋中的平均回收率为61.2%~105.7%,RSD为1.8%~17.6%,符合GB/T 27417-2017《合格评定 化学分析方法确认和验证指南》^[21]^中对回收率和精密度的要求。

**表4:** 鸡蛋、鹅蛋和鸭蛋样品中31种兽药的平均回收率和相对标准偏差(*n*=6)

Drug	Spiked/ (μg/kg)	Egg			Goose egg			Duck egg	
		Recovery/%	RSD/%		Recovery/%	RSD/%		Recovery/%	RSD/%
Levamisole	2	90.1	8.0		92.3	7.3		83.2	8.9
	5	81.8	10.2		85.3	6.4		88.0	7.3
	10	98.3	4.2		95.2	7.0		91.2	10.0
Trimethoprim	3	75.5	2.0		82.3	3.4		73.4	6.3
	10	78.5	1.8		90.2	7.6		84.2	5.5
	20	93.5	5.9		84.3	5.1		78.1	4.8
Tilmicosin	3	81.6	13.8		78.3	10.9		80.4	11.1
	10	93.6	15.2		85.4	7.5		86.8	9.1
	20	95.2	11.9		90.1	9.4		92.0	10.3
Danofloxacin	3	80.7	15.0		83.5	8.9		81.3	7.4
	10	84.7	6.1		76.7	11.2		77.8	6.6
	20	85.9	4.1		80.6	7.2		90.3	3.8
Enrofloxacin	3	73.5	11.3		70.7	10.6		69.3	9.8
	10	77.9	7.9		80.6	8.5		75.9	6.3
	20	81.6	2.8		78.2	6.7		82.3	8.0
Ciprofloxacin	3	69.4	12.3		72.3	8.9		68.5	11.8
	10	76.1	8.3		80.2	10.2		75.4	9.8
	20	82.0	5.5		78.9	7.7		83.7	5.6
Flumequine	3	83.3	7.2		90.5	8.4		87.4	9.4
	10	92.9	7.9		86.7	5.1		84.3	6.5
	20	102.3	3.8		95.6	6.3		96.0	5.8
Oxolinic acid	3	92.3	14.2		88.6	9.3		89.1	12.3
	10	86.9	8.7		96.3	11.5		86.7	8.9
	20	94.2	5.4		94.0	8.4		84.0	9.7
Sarafloxacin	3	71.7	15.2		67.5	13.3		66.2	16.4
	5	73.3	7.9		72.3	9.4		70.8	13.2
	10	81.6	3.2		78.2	6.8		74.3	8.9
Lomefloxacin	2	71.0	9.4		70.4	8.5		68.3	9.6
	4	75.3	11.9		69.8	6.8		73.6	7.3
	8	79.7	6.5		78.6	5.3		81.0	8.4
Norfloxacin	1	73.2	5.6		68.5	8.5		65.7	11.0
	2	78.1	3.4		73.8	4.8		68.4	7.6
	4	83.5	4.8		79.1	6.4		75.5	4.8
Ofloxacin	1	79.7	6.9		80.3	5.8		76.7	7.8
	2	85.3	5.0		84.5	3.3		80.6	5.0
	4	90.4	3.2		91.6	4.1		87.6	6.1
Pefloxacin	2	79.7	14.5		70.3	15.8		61.2	9.4
	4	79.8	12.8		74.9	10.4		70.5	8.7
	8	86.4	12.1		81.7	8.8		78.9	10.2
Doxycycline	10	69.2	14.7		70.3	12.3		66.8	13.4
	20	67.2	10.3		68.4	9.2		72.3	11.6
	40	73.5	6.3		75.9	10.5		74.0	8.7
Sulfadimidine	3	91.7	5.6		88.6	11.3		85.7	8.9
	10	86.8	11.9		97.5	8.7		90.6	3.4
	20	94.7	10.3		104.5	5.1		97.3	4.0
Sulfadiazine	3	68.7	10.6		68.5	12.3		70.4	10.6
	10	71.4	12.0		74.3	8.9		73.0	8.2
	20	73.9	6.4		81.5	6.0		82.0	7.7
Sulfathiazole	3	71.2	5.9		69.0	12.1		67.4	9.8
	10	76.7	13.9		73.1	8.7		75.0	7.9
	20	76.0	6.4		77.2	5.9		80.4	6.0
Sulfamerazine	3	74.1	16.3		76.5	13.5		72.3	12.3
	10	80.4	12.8		82.4	11.3		78.5	9.9
	20	85.4	9.4		90.1	8.8		85.7	6.8
Sulfameter	3	71.7	10.9		82.3	8.4		78.4	6.5
	10	84.1	13.4		87.6	7.6		80.2	4.5
	20	84.5	8.2		93.5	5.4		88.0	2.1
Sulfamonomethoxine	3	79.5	3.6		80.6	5.6		82.3	6.5
	10	88.1	10.1		90.3	6.0		79.1	4.8
	20	85.7	6.9		102.1	4.4		89.7	3.9
Sulfachloropyridazine	3	77.0	17.6		73.4	12.3		70.9	8.9
	10	78.2	14.7		82.1	8.7		82.3	7.4
	20	84.2	7.0		79.3	6.5		86.4	6.0
Sulfadoxine	3	83.3	8.2		78.3	7.8		83.1	8.2
	10	87.2	12.4		86.0	8.4		86.7	9.1
	20	92.0	5.7		95.5	6.0		92.4	5.5
Sulfadimethoxine	3	87.5	2.7		83.5	6.5		79.6	7.3
	10	86.9	10.5		90.5	4.8		85.4	8.0
	20	90.8	5.0		93.0	3.0		92.4	7.1
Sulfamethoxazole	3	72.3	6.9		70.3	8.4		72.6	6.8
	10	84.3	13.4		77.8	6.3		80.1	7.5
	20	89.0	4.0		86.7	5.0		84.5	6.0
Sulfaquinoxaline	3	61.7	8.2		63.4	10.5		65.0	8.2
	10	66.1	12.2		70.3	8.3		69.4	9.4
	20	75.6	4.1		76.0	5.6		75.7	7.0
Florfenicol-amine	3	85.3	7.4		83.5	6.1		81.0	8.0
	10	88.0	8.2		90.2	3.4		87.3	6.7
	20	90.1	6.1		89.2	4.6		92.2	3.1
Thiamphenicol	1	93.9	10.8		89.4	6.7		90.4	6.5
	10	99.7	10.3		95.0	5.2		93.5	4.7
	20	101.9	2.3		105.7	2.8		96.1	2.5
Florfenicol	1	86.6	11.4		83.4	9.3		85.6	10.3
	10	85.0	4.1		87.2	7.6		90.7	8.7
	20	96.9	1.9		93.5	5.9		91.5	6.2
Toltrazuril sulfone	5	63.4	10.6		65.3	8.6		67.4	9.7
	10	66.4	8.3		72.1	7.8		69.0	10.3
	20	68.5	8.0		74.0	5.5		65.2	7.3
Diclazuril	5	68.4	10.2		70.4	8.3		66.3	10.2
	10	74.3	7.0		68.5	9.9		72.1	8.7
	20	75.0	6.2		74.6	6.8		76.0	7.0
Salicylic acid	3	73.1	11.4		76.4	12.4		80.2	13.5
	10	83.5	3.1		73.1	13.0		82.1	11.7
	20	88.2	4.2		85.3	9.4		89.0	8.7

### 2.5 实际样品的测定

应用本工作建立的方法,对从超市和农贸市场采集的20批次鸡蛋、5批次鸭蛋和5批次鹅蛋进行检测。结果显示,1批次鸡蛋中检出恩诺沙星,结果为12.3 μg/kg,其MRM色谱图见图4。为验证本文方法,应用标准方法GB/T 21312-2007^[22]^,对检出恩诺沙星的阳性样品进行复检,结果为11.7 μg/kg。显示本工作建立的方法可有效用于禽蛋基质中相关药物的监测分析。

**图4:**
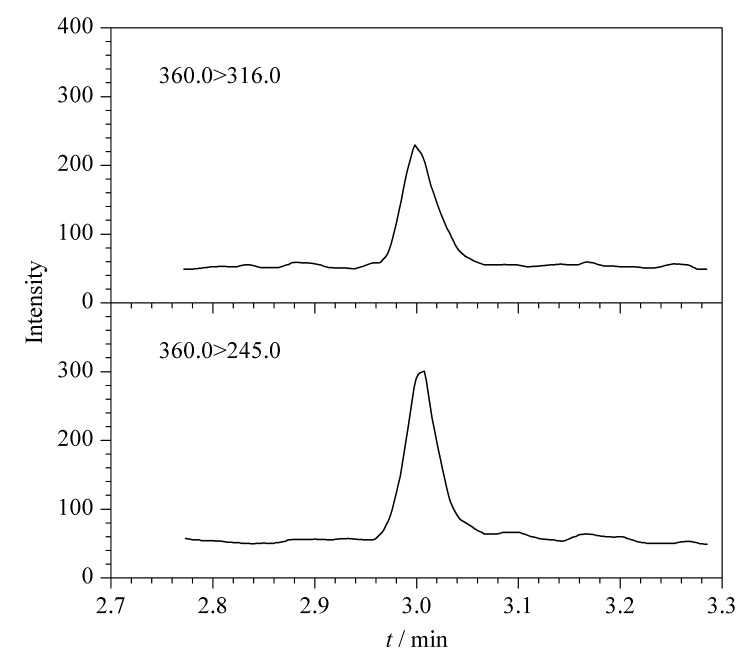
阳性样品中恩诺沙星的MRM色谱图

## 3 结论

本工作针对禽蛋样品,采用改良的QuEChERS法对样品进行处理,并结合超高效液相色谱-串联质谱,建立了31种产蛋期禁用兽药同时检测的方法。本方法操作简单,经济实用,检出限低,准确度高,重复性好,能满足GB 31650.1-2022标准规定的限量控制要求,可以为禽蛋质量安全监测提供有效可靠的技术支持。
